# Investigation of Long COVID Prevalence and Its Relationship to Epstein-Barr Virus Reactivation

**DOI:** 10.3390/pathogens10060763

**Published:** 2021-06-17

**Authors:** Jeffrey E. Gold, Ramazan A. Okyay, Warren E. Licht, David J. Hurley

**Affiliations:** 1 World Organization, Watkinsville, GA 30677, USA; 2 Department of Public Health, Kahramanmaraş Sütçü İmam University, Kahramanmaraş 46040, Turkey; razim01@gmail.com; 3 Warren Alpert Medical School of Brown University, Providence, RI 02903, USA; warren.licht@brownphysicians.org; 4 College of Veterinary Medicine, University of Georgia, Athens, GA 30602, USA; djhurley@uga.edu

**Keywords:** long COVID, post-acute COVID-19 syndrome, PACS, chronic COVID syndrome, Epstein–Barr virus reactivation, Epstein–Barr virus, EBV, SARS-CoV-2, COVID-19, coronavirus

## Abstract

Coronavirus disease 2019 (COVID-19) patients sometimes experience long-term symptoms following resolution of acute disease, including fatigue, brain fog, and rashes. Collectively these have become known as long COVID. Our aim was to first determine long COVID prevalence in 185 randomly surveyed COVID-19 patients and, subsequently, to determine if there was an association between occurrence of long COVID symptoms and reactivation of Epstein–Barr virus (EBV) in 68 COVID-19 patients recruited from those surveyed. We found the prevalence of long COVID symptoms to be 30.3% (56/185), which included 4 initially asymptomatic COVID-19 patients who later developed long COVID symptoms. Next, we found that 66.7% (20/30) of long COVID subjects versus 10% (2/20) of control subjects in our primary study group were positive for EBV reactivation based on positive titers for EBV early antigen-diffuse (EA-D) IgG or EBV viral capsid antigen (VCA) IgM. The difference was significant (*p* < 0.001, Fisher’s exact test). A similar ratio was observed in a secondary group of 18 subjects 21–90 days after testing positive for COVID-19, indicating reactivation may occur soon after or concurrently with COVID-19 infection. These findings suggest that many long COVID symptoms may not be a direct result of the SARS-CoV-2 virus but may be the result of COVID-19 inflammation-induced EBV reactivation.

## 1. Introduction

It has been reported that about 30% of coronavirus disease 2019 (COVID-19) patients experience long-term symptoms following resolution of acute disease [1]. These symptoms include fatigue, brain fog, sleep difficulties, arthralgia, pharyngitis, myalgia, headaches, fever, gastrointestinal upset, and skin rashes with a variety of presentations [2,3,4,5]. Long-term symptoms associated with COVID-19 are collectively known as long COVID. Long COVID has also been referred to as Post-Acute COVID-19 Syndrome (PACS) or chronic COVID syndrome (CCS) [6]. Long COVID has been associated with patients who have had subacute, mild, or severe COVID-19 cases [2].

Epstein–Barr virus (EBV) is a human gamma herpesvirus. It is known to have infected and generally become latent in more than 90% of the global population [7], including more than 95% of healthy adults [8]. It is found at high rates in every region of the world. This is due to both its lifelong persistence in the latent state and because of its intermittent recrudescence in many latently infected individuals [9]. Primary EBV infection is often asymptomatic when contracted in childhood. When primary infection occurs in adolescence or adulthood, however, it commonly results in infectious mononucleosis, an acute condition inducing massive lymphocytosis. EBV sometimes causes chronic infections or serially reactivated infections, in which it can efficiently infect both epithelial cells and B cells. EBV can also switch between lytic and latent phases of its life cycles in many patients [10].

EBV reactivation is most commonly identified in clinical practice using serological testing for the presence of EBV early antigen-diffuse (EA-D) IgG or EBV viral capsid antigen (VCA) IgM [11,12,13]. EBV VCA IgM is usually only detectable during the acute early stage of primary or reactivated EBV infection. In contrast, EBV EA-D is more likely to be detected only during the later chronic stage of EBV infection [14]. Therefore, multiple testing methods are required to accurately detect EBV reactivation.

A variety of clinical manifestations have been associated with EBV reactivation. These include fatigue, psychoneurosis/brain fog, sleep disturbance, arthralgia, pharyngitis, myalgia, headaches, fever, gastrointestinal complaints, and various skin rashes [11]. We observed that many symptoms attributed to long COVID are the same as, or very similar to, those that have been associated with EBV reactivation.

Our aim in this retrospective study was to first determine long COVID prevalence among COVID-19 patients surveyed and, subsequently, to determine if there was evidence of a relationship between occurrence of long COVID symptoms and EBV reactivation among the subjects recruited from those surveyed.

## 2. Results

### 2.1. Long COVID Prevalence

An analysis of the 185 subjects who applied to our study, all of whom provided evidence of confirmed COVID-19 infections, revealed that 30.3% (56/185) reported unabating long COVID symptoms at least 30 days after testing positive for COVID-19. This group included 13 subjects who had initially asymptomatic COVID-19 infections, among which 30.8% (4/13) went on to develop long COVID symptoms a few weeks after testing positive for COVID-19. Applicants were not aware of our intent to study long COVID. This blinding of the study subjects limited self-selection bias in the reporting of long COVID symptoms. The prevalence rate we observed was similar to the 30% prevalence rate observed in a University of Washington survey of 177 COVID-19 subjects followed for several months after their initial COVID-19 diagnosis [1].

### 2.2. Analysis of Primary (Long-Term) Study Participants

We found that 66.7% (20/30) of long-term long COVID subjects versus 10% (2/20) of long-term control subjects were positive for EBV reactivation based on positive titers for EBV EA-D IgG or EBV VCA IgM. The difference in the fraction showing reactivation between the groups was found to be significant (*p* < 0.001, Fisher’s exact test). Eighteen of the long-term long COVID subjects were positive for EBV EA-D IgG, one of which was also positive for EBV VCA IgM. Two additional long-term long COVID subjects were positive for EBV VCA IgM but not EBV EA-D IgG. Notably, two long-term long COVID subjects who were positive for EBV reactivation had asymptomatic COVID-19, with long COVID symptoms developing a short time later. The two subjects in the long-term control group positive for EBV reactivation were positive for EBV EA-D IgG only. Complete EBV antibody titer assessments of long-term study subjects appear in Appendix A (Table A1 and Table A2).

### 2.3. Analysis of Secondary (Short-Term) Study Participants

As those subjects enrolled as participants in the primary study were at least 90 days post-diagnosis of COVID-19, we also examined a secondary population who were between 21 to 90 days (short-term) post-diagnosis of COVID-19. We observed a similar level of EBV reactivation among these short-term subjects. We found that 66.7% (6/9) of short-term long COVID subjects showed evidence of EBV reactivation based on positive titers for EBV EA-D IgG or EBV VCA IgM. Among the short-term control subjects, 11.1% (1/9) tested positive for EBV reactivation based on positive titers for EBV EA-D IgG or EBV VCA IgM. The difference in the fraction showing evidence of reactivation between the short-term long COVID and control subjects was significant (*p* = 0.05, Fisher’s exact test). Six short-term long COVID subjects tested positive for EBV EA-D IgG, one of whom also tested positive for EBV VCA IgM. The single subject in the short-term control group indicating EBV reactivation was positive only for EBV EA-D IgG. Complete EBV antibody titer assessments of short-term study subjects appear in Appendix A (Table A3 and Table A4).

### 2.4. Assessment of EBV EA-D IgG, EBV VCA IgG, and EBNA-1 IgG Titers in All Subjects

**Figure 1:**
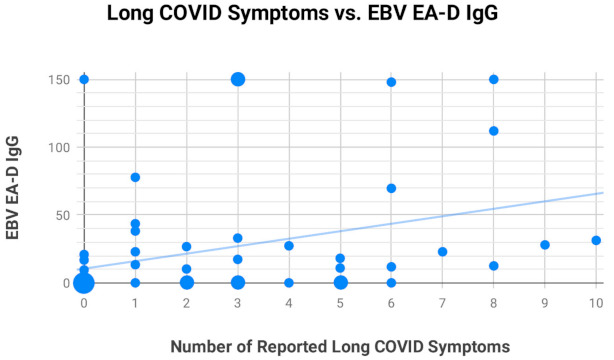
The relationship between EBV early antigen-diffuse (EA-D) IgG antibody titers and reported long COVID symptoms in the 68 subjects making up the primary and secondary groups was significant (r = 0.34, *p* < 0.001).

**Figure A1:**
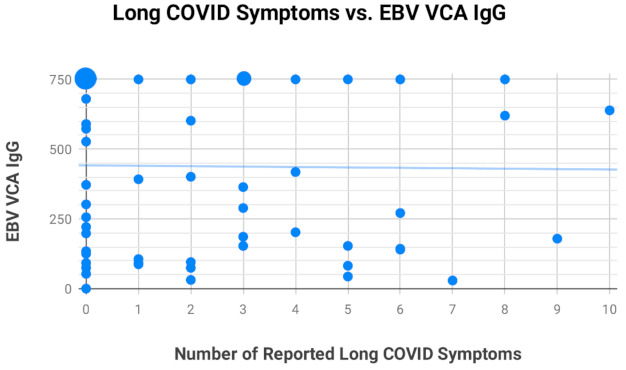
The relationship between EBV VCA IgG antibody titers and reported long COVID symptoms in the 68 subjects making up the primary and secondary groups was not significant (r = −0.014, *p* = 0.75).

**Figure A2:**
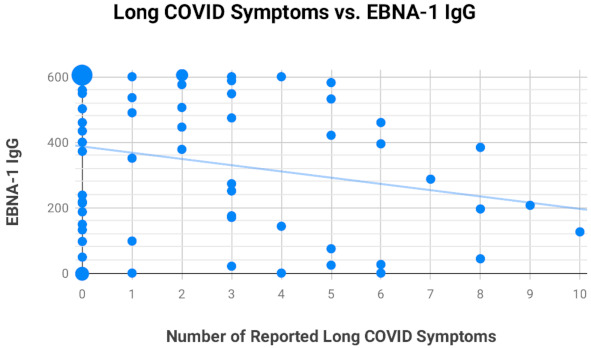
The relationship between EBV nuclear antigen 1 (EBNA-1) IgG antibody titers and reported long COVID symptoms in the 68 subjects making up the primary and secondary groups was not significant (r = −0.23, *p* = 0.09).

We did three analyses comparing EBV EA-D IgG, EBV VCA IgG, and EBNA-1 IgG antibody titer values against the number of long COVID symptoms reported by each of the 68 subjects making up our primary and secondary study groups to see if any significant relationships were observed. Only EBV EA-D IgG (Figure 1) demonstrated a significant relationship with the number of reported long COVID symptoms (r = 0.34, *p* < 0.001). Neither EBV VCA IgG (Figure A1) nor EBNA-1 IgG (Figure A2), shown in Appendix A, showed a statistically significant relationship with the number of long COVID symptoms.

### 2.5. Most Frequently Reported Symptoms

**Figure 2:**
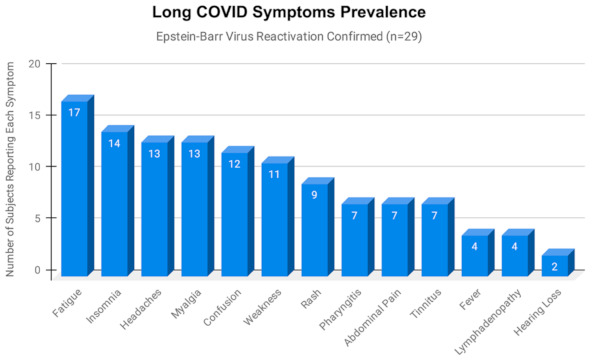
The number of subjects reporting each of 13 clinical manifestations of long COVID, as reported by the 29 subjects from both our long-term and short-term long COVID groups who tested positive for Epstein–Barr virus (EBV) reactivation. The percent of subjects with EBV reactivation reporting each symptom was: fatigue 58.6%, insomnia 48.3%, headaches 44.8%, myalgia 44.8%, confusion/brain fog 41.4%, weakness 37.9%, rash 31.0%, pharyngitis 24.1%, abdominal pain 24.1%, tinnitus 24.1%, fever over 101° F 13.8%, neck lymphadenopathy 13.8%, and mild-to-moderate hearing loss 6.9%.

**Figure 3:**
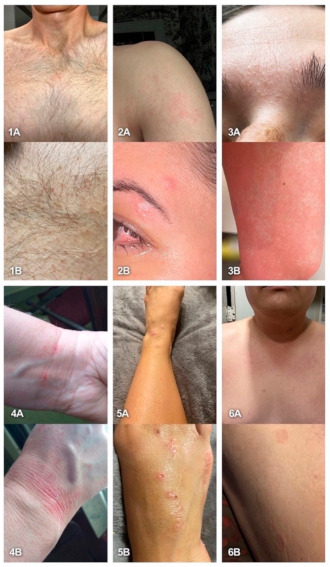
Skin manifestations of six long COVID subjects positive for EBV reactivation (two photos of each subject).

**Figure 4:**
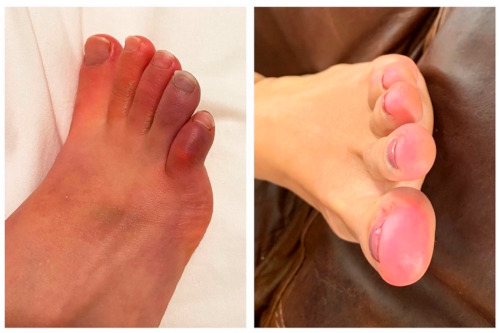
One subject experienced COVID toes at four months and another at nine months after testing positive for Coronavirus disease 2019 (COVID-19).

The most frequently reported symptoms among those who were positive for EBV reactivation from both our long-term and short-term long COVID groups were fatigue, insomnia, headaches, myalgia, and confusion (Figure 2). Seven subjects in the long-term long COVID group experienced tinnitus and/or some hearing loss. Seven subjects in the long-term long COVID group and two subjects in the short-term long COVID group with EBV reactivation experienced frequent skin rashes (Figure 3), including two with COVID toes (Figure 4), a condition associated with some COVID-19 cases [15]. No formal statistical assessment of the frequency of these long COVID manifestations was attempted; our findings were observational only.

## 3. Discussion

EBV can be serially reactivated as the result of a variety of stressor events [16]. Stress levels have been linked to the duration and intensity of reactivated EBV infections and variations of the steady-state expression of latent EBV [17,18].

Chen et al. (2021) of Remnin Hospital at Wuhan University in Wuhan, Hubei province, China were the first to document finding EBV reactivation in COVID-19 patients during the acute phase. They found that 55.2% of hospitalized COVID-19 patients between 9 January 2020 and 29 February 2020 with serological confirmation of past EBV infection also tested positive for EBV VCA IgM, indicating EBV reactivation within two weeks of testing positive for SARS-CoV-2 [19].

Paolucci et al. (2020) tested 104 COVID-19 patients, 42 in an intensive care unit (ICU) and 62 in a sub-intensive care unit (SICU) in Italy and observed EBV reactivation in 95.2% (40/42) of the ICU patients and in 83.6% (51/61) of the SICU patients. They further determined that the median EBV DNA level in ICU patients was significantly higher than that of SICU patients [20]. A similar study in France found evidence of EBV reactivation in 82% (28/34) of COVID-19 ICU patients. Further, they found EBV reactivation to be associated with longer median ICU stays (15 days versus 8 days, *p* < 0.05) [21].

Lehner et al. (2020) ran EBV and cytomegalovirus (CMV) DNA tests on COVID-19 patients in the Medical ICU at the Medical University Innsbruck, Austria and found that 78% of the COVID-19 patients they tested with respiratory failure requiring invasive ventilation had evidence of EBV viremia [22]. However, CMV viremia was not found to be any more common in COVID-19 patients than in non-COVID-19 patients.

While a limitation of our study is that we were not able to pinpoint the exact timing of EBV reactivation in the subjects we studied, given that we found similar reactivation frequencies in both long-term and short-term long COVID subjects, this indicates a likelihood that EBV reactivation occurs early in SARS-CoV-2 infection. Early EBV reactivation has been previously documented in several studies of COVID-19 ICU patients [20,21,22,23].

More than 90% of adults carry antibodies indicating past EBV infection. These infections most often occur from childhood through the early twenties. When primary infection occurs in the teens or later, infectious mononucleosis can be the clinical result. In the United States, there is a recognized racial disparity in the typical age of primary EBV infection. Seroprevalence of prior EBV infection in those under age 20 is much higher among Hispanic Americans (85.4%, 95% CI 83.1–87.8%) and Non-Hispanic Blacks (83.1%, 95% CI 81.1–85.1%) than in Non-Hispanic Whites (56.9%, 95% CI 54.1–59.8%). The greatest disparity is observed through age 14 [24].

Two tests used to detect prior EBV infection in clinical practice, EBV VCA IgG and EBV nuclear antigen 1 (EBNA-1) IgG, return a positive result soon after primary EBV infection and typically remain positive for life. A positive result for both is typically indicative of past EBV infection. A positive result for EBV VCA IgG, but not for EBNA-1 IgG, may also indicate a past EBV infection in cases where patients were immunosuppressed or when individuals never produced EBNA-1 IgG at all [25].

**Figure 5:**
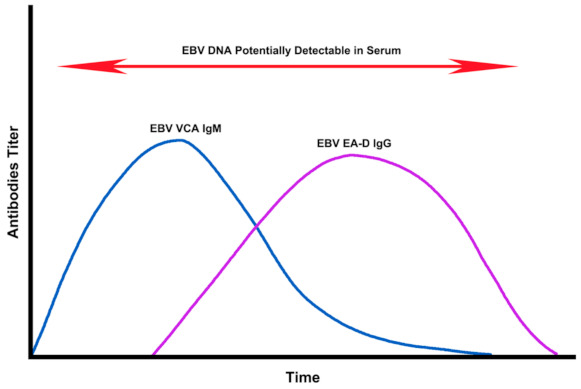
The dynamics of EBV viral capsid antigen (VCA) IgM titers, EBV early antigen-diffuse (EA-D) IgG titers, and serum EBV DNA over time after EBV infection or reactivation [14,26,27,28].

EBV reactivation is typically identified by testing for the presence of EBV EA-D IgG or EBV VCA IgM [11,12,13]. EBV reactivation can also identified by testing for the presence of circulating EBV DNA utilizing a serum EBV DNA quantitative real-time polymerase chain reaction (PCR) test. EBV EA-D IgG, EBV VCA IgM, and serum EBV DNA are often detectable at separate times during the course of primary or reactivated EBV infection (Figure 5), requiring the use of multiple testing methods to accurately determine if an individual is positive for EBV reactivation. While EBV DNA is most often detectable during the acute early stage of primary or reactivated EBV infection, EBV DNA can also be detected at other stages of EBV infection, depending on the sensitivity of the quantitative PCR tests used [26,27], as well as the frequency of testing.

In order to investigate how serum EBV DNA tests may help clinicians further identify patients with EBV reactivation, we utilized a commercial EBV DNA quantitative real-time PCR test on all long-term and short-term long COVID subjects who tested negative for both EBV EA-D IgG and EBV VCA IgM. EBV DNA testing identified two additional subjects in the long-term long COVID group showing evidence of EBV reactivation based on the presence of circulating EBV DNA (465 copies/mL and 481 copies/mL) and one additional short-term long COVID subject positive for EBV reactivation based on the presence of circulating EBV DNA (578 copies/mL). The commercial assay source we used indicated that a minimum of 200 copies/mL constituted a positive test. Once those positive for EBV DNA were added to the dataset of subjects already found to be positive for EBV reactivation, we found that 73.3% (22/30) of long-term long COVID subjects and 77.8% (7/9) of short-term long COVID subjects showed evidence of EBV reactivation.

EBV reactivation is known to induce a diverse set of rashes and skin lesions that include urticaria [29], granuloma annulare [30], folliculitis [31], cryoglobulinemia [32,33], and Raynaud’s phenomenon [34], which resembles COVID toes [35]. One of the earliest documented cases of COVID toes was described in Madrid, Spain by Nirenberg et al. in April 2020 in a 16-year-old female who had a coinfection of EBV and COVID-19 [36]. A wide variety of skin manifestations were reported by nine subjects among our study groups who tested positive for EBV reactivation, including two who experienced COVID toes: one at four months and the other at nine months post-diagnosis of COVID-19.

EBV has been associated with tinnitus and hearing loss [37,38]. Tinnitus is a common long COVID symptom and was reported by seven subjects in our study groups who tested positive for EBV reactivation. Mild-to-moderate hearing loss was reported by two subjects in our study groups who tested positive for EBV reactivation. Unexplained hearing loss and tinnitus tied to SARS-CoV-2 have been documented in various case reports and small studies [39,40,41].

In addition to the more common manifestations described earlier, EBV reactivation has also been associated with cardiovascular, hematological, and neurological complications [42]. EBV reactivation has been reported to play a role in the pathogenesis of myocarditis [43], inflammatory cardiomyopathy [44], and acute myocardial infarction [45]. EBV-associated multisystem failure has been documented to also result in acute liver injury, kidney injury, respiratory failure, and hemolytic anemia in immunocompetent patients [46]. EBV is also associated with a number of lymphoid and epithelial tumors [47]. While rare, given that EBV reactivation has been associated with many serious clinical manifestations, further study would be prudent to determine if any of these become more frequent in COVID-19 patients.

Currently, there are no pharmaceuticals licensed to specifically treat EBV reactivation. Some anti-DNA viral agents have been used to attempt to reduce the viral load in reactivation of herpes viruses. Some level of efficacy in the management of EBV disease has been demonstrated when using these drugs. Extended administration of valacyclovir is known to reduce the frequency of EBV-infected B cells and has been theorized as a treatment to eradicate EBV from the body [48]. Spironolactone has been found in vitro to inhibit EBV VCA synthesis and capsid formation [49]. Spironolactone is also being studied as a potential therapeutic for SARS-CoV-2 infection itself [50,51]. Further evidence that EBV may be contributing to COVID-19 disease comes from a clinical study in Wuhan. This study showed that treatment with ganciclovir, an anti-herpesvirus drug that blocks the replication of EBV, reduced the risk of death in patients with severe disease [52].

An awareness of the associations between SARS-CoV-2 and EBV reactivation creates new opportunities for long COVID diagnosis, management, and possible treatments. We believe that it would be prudent to determine if patients who have tested positive for COVID-19 have evidence of EBV reactivation, whether showing classical acute disease or not. If they do show signs of EBV reactivation, then it would be clinically appropriate to monitor for the development of known EBV disease manifestations, particularly those that are shared with the long COVID complex.

Our study opens up several new avenues for future research. Long COVID patients’ antibody responses to CMV could be studied alongside EBV to determine whether CMV may also be reactivated in some cases of long COVID. Given the numerous skin manifestations observed in long COVID cases, it would be interesting to investigate if EBV viral loads can be observed in enriched T cells in such cases. Another area for study would be to test peripheral blood mononuclear cells (PBMCs) of long COVID patients to determine if the cellular immune compartment or serum factors facilitate EBV reactivation.

In conclusion, our results indicate that approximately 30% of COVID-19 patients report long COVID-like symptoms after acute disease. EBV reactivation may occur soon after or concomitantly with COVID-19 infection, including after initially asymptomatic infections. The SARS-CoV-2 virus may stimulate sequalae involving other infectious agents that contribute to many long COVID symptoms. Thus, it is worth considering that a portion of long COVID symptoms may be the result of COVID-19 inflammation-induced EBV reactivation.

## 4. Materials and Methods

### 4.1. Study Design

**Figure 6:**
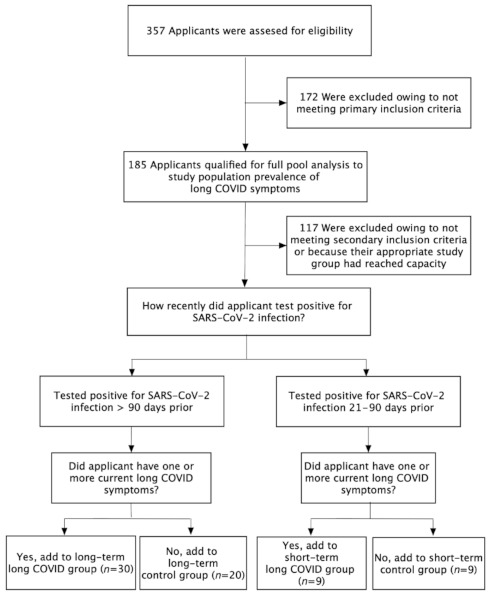
Primary inclusion: subjects were included only if they provided the requested background, including documentation of their COVID-19 diagnosis, most often in the form of SARS-CoV-2 polymerase chain reaction (PCR) results. Secondary inclusion: subjects were included only if between the ages of 21–74, with no exclusionary health conditions.

We screened 357 applicants (Figure 6) using a Health Insurance Portability and Accountability Act (HIPAA)-compliant online form under which informed consent was obtained. As an assurance of confidentiality, only one investigator performed recruitment, data collection, and validation. Patient data was kept in a secure database that was used only in coded form (to remove all patient identifiers) before the analysis of the data by the research team. Thus, all patient records, test data, and the identity of the subjects submitting photos of skin manifestations were kept by a single source.

### 4.2. Patient Recruitment

Subjects were chosen from applicants who responded to online advertisements seeking recovered COVID-19 patients for this study. Potential subjects were only aware that we wished to collect data related to their experience with COVID-19 disease, thus limiting self-selection bias. Each applicant was required to upload documentation of their COVID-19 medical history. This included copies of COVID-19 test results and hospitalization records, as well as completing an in-depth online survey in which they provided details related to their COVID-19 symptoms and outcomes on the HIPAA-compliant form. Follow-up was done by one investigator to verify that each subject met the study criteria and to allow subjects demonstrating skin manifestations to provide images of such for the record and to be kept in blinded files for evaluation by the remaining researchers. Subjects in the study were selected randomly from all who applied and were a match to the criteria for any of the study groups. Applicants were excluded if under 21 years of age, over 74 years of age, if they were pregnant, had been given a COVID-19 vaccine, or had symptoms similar to long COVID prior to testing positive for COVID-19. The selection process continued until 68 qualified subjects were added to the study pool. These subjects provided serological samples at a clinical laboratory to be tested for the relevant EBV parameters. The selection of applicants and serological testing was conducted from 11 December 2020 through 11 February 2021. A small stipend to help defray costs associated with providing records and blood samples was available to subjects.

### 4.3. Primary and Secondary Study Groups

We divided subjects into four groups: two primary (long-term) study groups and two secondary (short-term) study groups. The “long-term long COVID group” consisted of 30 subjects; all had tested positive for COVID-19 at least 90 days prior to being enrolled, and all reported one or more of the long COVID symptoms utilized for this study. The “long-term control group” consisted of 20 subjects; all had tested positive for COVID-19 at least 90 days prior to enrollment, and none reported any of the long COVID symptoms we were assessing. The “short-term long COVID group” consisted of 9 subjects; all had tested positive for COVID-19 21–90 days prior to enrollment, and all reported one or more of the long COVID symptoms utilized for this study. The “short-term control group” consisted of 9 subjects; all had tested positive for COVID-19 21–90 days prior to enrollment, and none reported any of the long COVID symptoms we were assessing.

Long COVID subjects were those that reported one or more of the following unabating symptoms after recovering from initial SARS-CoV-2 infection: fatigue, insomnia, headaches, myalgia, confusion/brain fog, weakness, rash, pharyngitis, abdominal pain, tinnitus, fever over 101° F, neck lymphadenopathy, or mild-to-moderate hearing loss.

The age and the sex of long-term long COVID subjects versus long-term control subjects (Table 1) were commensurate with each other. Additionally, the geographic distribution of participants across United States census regions (Table 2) gave us no reason to conclude that the distribution was unequal. Therefore, we believe there was little chance significant bias was introduced by the selection process for study applicants.

### 4.4. Assessments

All study participants volunteered to provide blood samples through a clinical reference laboratory (Quest Diagnostics, Secaucus, NJ, USA). The samples were tested for EBV VCA IgM, EBV VCA IgG, EBNA-1 IgG, and EBV EA-D IgG, as shown in Appendix A (Table A1, Table A2, Table A3 and Table A4). Subjects with long COVID symptoms who did not test positive for EBV VCA IgM or EBV EA-D IgG were also tested for EBV DNA with a quantitative real-time PCR test with a linear range of 200–2,000,000 copies/mL.

EBV antibodies were measured using a LIAISON® Analyzer (DiaSorin, Centralino, Italy) to measure the chemiluminescence from a commercially available immunoassay (CLIA) for the qualitative determination of IgG and IgM antibodies in human serum specimens. The method for qualitative determination of specific IgG and IgM antibodies to EBV was a competitive (indirect) CLIA. The principal components of the EBV VCA IgG and EBV VCA IgM tests were magnetic particles coated with VCA p18 synthetic peptide, BSA, and a phosphate buffer containing < 0.1% sodium azide. The principal components of the EBNA-1 IgG tests were magnetic particles coated with EBNA-1 synthetic peptide, BSA, and a phosphate buffer containing < 0.1% sodium azide. The principal components of the EBV EA-D IgG tests were magnetic particles coated with EA-D polypeptide (obtained in *E. coli* by recombinant DNA technology), BSA, and a phosphate buffer containing < 0.1% sodium azide. The EBV DNA quantitative real-time PCR test was developed in-house, and its analytical performance was validated by Quest Diagnostics.

When EBV VCA IgM can be detected but EBNA-1 IgG cannot, this generally indicates primary EBV infection or EBV reactivation. When EBV VCA IgM and EBNA-1 IgG are both detectable, this generally indicates EBV reactivation. EBV EA-D IgG is generally only detected in patients with either primary infection or EBV reactivation. Thus, testing for the presence of EBV VCA IgM or EBV EA-D IgG has been commonly used to detect EBV reactivation [11,12,13]. Some reactivation cases missed by titer tests can be detected through serum testing for the presence of EBV DNA circulating following viral release during recrudescence. In our study, a subject was classified as having EBV reactivation if they exceeded any of these threshold values: EBV VCA IgM > 39.99 U/mL, EBV EA-D IgG > 9.99 U/mL, or EBV DNA quantitative, real-time PCR > 199 copies/mL.

### 4.5. Review of Photographs

Subjects with skin manifestations of long COVID provided photographs of their own skin problems. The investigators participating in this study reviewed the blinded images of skin manifestations provided by the collecting investigator to determine if they were consistent with skin manifestations that were reported in other long COVID patients and people with recurring EBV reactivation. A consensus was found that the images presented here represented lesions described for both conditions. No formal statistical assessment of these manifestations was attempted, and the data was provided descriptively.

### 4.6. Statistical Analysis

We used Fisher’s exact test to compare reactivation rates in the long COVID groups versus the control groups. All calculations of statistical significance and power analysis related to sample size were performed using R (The R Foundation for Statistical Computing, version 4.0.3, Vienna, Austria). A value of *p* ≤ 0.05 was considered to indicate statistical significance. We planned for a sample size of 30 in the long-term long COVID group and a sample size of 20 in the long-term control group, yielding 99% power using an expected difference of 80% versus 20%, respectively. (Assumptions: two-sided Z-test applied to the arcsine transformation of the proportions, alpha = 0.05.) All power analyses (for sample size) and calculations of statistical significance were completed independently by statisticians Nayak Polissar, PhD and Ljubomir Miljacic, PhD, MS (Seattle, WA, USA).

## Figures and Tables

**Table 1:** Characteristics of participants in the primary (long-term) study groups (plus–minus values are means ± SD).

Characteristic	Long-Term Long COVID Group	Long-Term Control Group
Mean Age	43.8 ± 13.4	43.9 ± 13.7
≤40 years—no. (%)	14 (46.7)	9 (45.0)
>40 years—no. (%)	16 (53.3)	11 (55.0)
Female—no. (%)	23 (76.7)	14 (70.0)
Male—no. (%)	7 (23.3)	6 (30.0)

**Table 2:** Geographic distribution of participants in the primary (long-term) study groups ^1^.

United States Census Region	Long-Term Long COVID Group	Long-Term Control Group
Region 1: Northeast—no.	10	4
Region 2: Midwest—no.	5	3
Region 3: South—no.	5	6
Region 4: West—no.	10	7

^1^ We tested the null hypothesis that the distribution of subjects in each of the groups was not different using Fisher’s exact test (*p* = 0.65).

## Data Availability

A complete database of deidentified individual subject data for all measured parameters is available online at Dryad: https://doi.org/10.5061/dryad.c866t1g67.

## Appendix A. Epstein–Barr Virus Antibody Panel Results

**Table A1:** Long-Term long COVID group Epstein–Barr virus antibody panel results (- = negative).

ID	EBV EA-D IgG	EBV VCA IgM	EBV VCA IgG	EBNA-1 IgG
06966	-	81.4	>750	>600
13444	148	-	143	460
13459	-	-	202	143
15170	-	-	>750	532
15366	10.2	-	401	576
15994	-	-	>750	548
16370	69.7	-	>750	-
16381	-	-	153	74.4
16968	22.9	-	>750	>600
16979	>150	-	>750	273
17050	-	-	>750	351
17113	77.8	69.1	106	490
17332	13.4	-	87.2	97.8
17706	-	-	31.1	378
35143	18.1	-	81.9	582
35145	>150	-	620	196
35188	26.7	-	602	446
35946	112	-	>750	384
83584	-	67.1	364	588
83821	>150	-	153	21
83860	-	-	>750	>600
83893	28	-	179	207
83925	-	-	95	506
83929	10.9	-	43.4	24.1
83938	31.3	-	639	126
87629	-	-	>750	251
87634	-	-	74.4	>600
87987	17.3	-	289	175
87999	33	-	>750	474
94332	22.9	-	29.1	287

**Table A2:** Long-term control group Epstein–Barr virus antibody panel results (- = negative).

ID	EBV EA-D IgG	EBV VCA IgM	EBV VCA IgG	EBNA-1 IgG
13481	-	-	198	372
16169	-	-	134	149
16199	16.8	-	>750	502
16710	-	-	680	>600
16715	-	-	573	187
16721	-	-	125	>600
16755	-	-	-	-
16832	-	-	198	48.7
17783	-	-	91.9	>600
17804	-	-	302	>600
83740	-	-	>750	460
83859	-	-	53	559
83911	-	-	>750	400
83924	-	-	74.6	132
83966	-	-	>750	589
84549	-	-	129	434
85068	9.51	-	>750	>600
87612	-	-	>750	96.6
87994	>150	-	>750	>600
93070	-	-	>750	238

**Table A3:** Short-term long COVID group Epstein–Barr virus antibody panel results (- = negative).

ID	EBV EA-D IgG	EBV VCA IgM	EBV VCA IgG	EBNA-1 IgG
13963	-	-	>750	421
16378	43.6	-	92.3	536
16555	-	-	271	26.2
16717	38.2	-	392	-
16750	27.3	-	418	-
17106	-	-	>750	>600
17323	>150	-	186	170
17387	12.5	47.5	>750	43.7
17390	11.8	-	140	395

**Table A4:** Short-term control group Epstein–Barr virus antibody panel results (- = negative).

ID	EBV EA-D IgG	EBV VCA IgM	EBV VCA IgG	EBNA-1 IgG
07506	-	-	221	214
09092	-	-	>750	549
15509	20.9	-	372	>600
16406	-	-	>750	-
16642	-	-	590	218
16770	-	-	256	>600
92742	-	-	>750	-
93074	-	-	527	>600
94266	-	-	>750	-
